# Long-Term Effects of Extracorporeal Shock Wave Therapy on Breast Cancer-Related Lymphedema

**DOI:** 10.3390/jcm11226747

**Published:** 2022-11-15

**Authors:** Jong-Hwa Lee, Sang-Beom Kim, Kyeong-Woo Lee, Won-Wook Ha

**Affiliations:** Department of Physical Medicine and Rehabilitation, Dong-A University College of Medicine, Busan 49201, Republic of Korea

**Keywords:** breast cancer related lymphedema, extracorporeal shockwave therapy, fibrosis, long-term effect

## Abstract

Extracorporeal shock wave therapy (ESWT) can reduce breast cancer-related lymphedema (BCRL). However, evidence of the long-term effectiveness of ESWT on BCRL is sparse. The aim of the study was to investigate whether ESWT has long-term effects on BCRL. We enrolled patients with stage 2 lymphedema. The 28 female patients were randomly divided into the ESWT group (n = 14) and the control group (n = 14). ESWT was applied thrice a week for a total of 3 weeks with an intensity of 0.056 to 0.068 mJ/mm^2^ and a frequency of 4 Hz. Complex decongestive therapy (CDT) was applied in both groups. The arm circumference, fluid volume, ratio of water content, and skin thickness were measured. Patients were evaluated at before treatment, 3 weeks after ESWT completion, and 3 months post-ESWT completion. The ESWT group, the circumference of the whole arm, volume, ratio of water content, QuickDASH score, and skin thickness showed statistically significant improvement at 3 weeks and 3 months post-treatment. When comparing the changes in measurement between the two groups at 3 weeks and 3 months post-treatment, ESWT group showed statistically significant improvement in circumference (cm) below the elbow, ratio of water content and skin thickness at 3 weeks and 3 months post treatment. Overall, ESWT improved lymphedema in patients with stage 2 BCRL, and the effects persisted for at least 3 months. Therefore, ESWT may be an additional treatment method for patients with lymphedema.

## 1. Introduction

Lymphedema is a chronic and progressive condition caused by abnormalities or damage to the lymphatic system. It is characterized by an abnormal increase in tissue protein levels, edema, chronic inflammation, and fibrosis. Secondary lymphedema can be caused by factors related to lymphatic stasis, such as tumor lymph node infiltration, lymph node dissection, radiation therapy, trauma, and infection [1]. The incidence of breast cancer-related lymphedema is reported to be about 21.4% [2]. Lymphedema is classified into four stages. Stage 0 is a latent or asymptomatic state in which swelling is not evident despite disturbances in lymphatic transport. In stage 1, fluid with a relatively high protein content accumulates, but edema subsides when the affected extremity is elevated. In stage 2, the edema does not subside by elevating the affected extremity, and pitting edema appears. In the later stages, excessive subcutaneous fat deposition and fibrosis occur, and pitting edema may not be evident; stage 3 does not present with pitting edema, but changes, including an elephant leg appearance, thickening of the skin, fat deposition, and fibrosis, occur [3]. The most common method for treating lymphedema is complex decongestive therapy (CDT). CDT is divided into two phases: phase 1 is an intensive treatment period that consists of skin care, manual lymphatic drainage, application of multilayer compression bandage, and exercise. When edema subsides, phase 2 is initiated, which focuses on self-care through application of daytime elastic sleeves or stocking compression, night bandage, and continued exercise [4]. CDT is the gold standard therapy for lymphedema and its effectiveness has been proven in all stages. CDT is associated with volume reduction in the affected limb as well as improved quality of life [5]. However, the effect of CDT may vary depending on factors such as the patient’s character and the therapist’s ability [6,7,8].

Extracorporeal shock wave therapy (ESWT) is a widely used physical therapy modality. Generally, ESWT emit acoustic waves (shock waves), which can transmit energy and propagate through the tissue. Shockwaves can trigger interstitial and extracellular responses, producing many beneficial effects, such as pain relief, angiogenesis, protein biosynthesis, cell proliferation, nerve and chondroprotection, and disruption of calcium deposits in musculoskeletal structures. The combination of these effects may lead to tissue regeneration, significant pain relief, and functional improvement of the damaged tissue [9]. Similarly, several studies have demonstrated that low-energy ESWT aids tissue regeneration by increasing stem cell activity, promoting endothelial neovascularization, modulating inflammation, relieving pain, and preventing soft tissue fibrosis [10,11,12]. In animal studies, ESWT activated vascular endothelial growth factor (VEGF) and fibroblasts, thereby promoting lymphatic neovascularization [13]. The effectiveness of ESWT in patients with breast cancer-related lymphedema (BCRL) is well-established [14,15,16]. However, evidence of the long-term effectiveness of ESWT on BCRL still remains sparse. Therefore, to the aim of this study is to investigate the long-term effects of ESWT on BCRL.

## 2. Materials and Methods

### 2.1. Study Population

Patients who underwent mastectomy after diagnosis of breast cancer and visited the Rehabilitation Medicine Department for lymphedema treatment were recruited for this study. To objectively confirm lymphedema, patients were characterized in accordance to the International Lymph Society (ISL) criterion, wherein stage 2 lymphedema was defined by fibrosis and no reduction in swelling following limb elevation. A difference of 2 cm in the circumference of both arms measured at the elbow, wrist, metacarpophalangeal joints, and 10 cm above and below the elbow with a volume difference of 200 mL between both arms as verified by immersion were included in the study. All patients’ positive stemmer sign was confirmed by the physiatrist.

Patients with acute and chronic inflammation, bilateral lymphedema, or metastases were excluded. Patients who had previously undergone CDT and had an underlying history that could affect lymphedema (e.g., chronic liver disease, chronic renal disease, congestive heart failure, other endocrine system problems, etc.) were also excluded. Finally, 28 patients were randomly assigned to either the ESWT group or control group. Block randomization was done using a computerized program. The total study period was from November 2020 to January 2022. Dong-A University Hospital Institutional Review Board (DAUHIRB) reviewed this study and approved (DAUHIRB-22-099).

### 2.2. Methods

In the ESWT group, shock waves were applied using an Electromagnetic type Dornier AR2 (Dornier MedTech GmBH, Wessling, Germany) thrice a week for a total of 3 weeks. The extracorporeal shock wave intensity ranged from 0.056 to 0.068 mJ/mm^2^ and a frequency of 4 Hz and was applied 1000 times to the most fibrotic area of the forearm and 1500 times to the lymph nodes of the elbow, upper arm, forearm, and hand. CDT, such as manual lymphatic massage, elastic bandage therapy, exercise, and skin care, was performed for both the ESWT (n = 14) and control (n = 14) groups.

CDT consisted of a three-week decongestive phase followed by a maintenance phase. The decongestive phase consisted of manual lymphatic drainage (MLD), compression bandaging, and skin care, and each daily session lasted 60 min. Treatment was performed by the same physical therapist. Additionally, during this period, patients were educated on how to perform MLD, exercise, bandage, and skin care on their own. The maintenance phase consisted of daily self-administered MLD, exercise, self-applied bandage and skin care.

### 2.3. Assessment

Patients were evaluated thrice: before treatment, 3 weeks after ESWT completion, and 3 months post-ESWT completion. The circumference, fluid volume, ratio of water content, and skin thickness of both extremities were measured. The circumference of both arms was measured at the elbow, wrist, metacarpophalangeal joints, and 10 cm above and below the elbow. Volume measurement was based on Archimedes’ principle. The entire arm, from the hand to the axilla, was immersed in water. The change in water volume was then recorded, and both the affected and unaffected extremities were compared.

An InBody S10 (Biospace Co., Seoul, South Korea) body composition analyzer was used to compare the ratio of extracellular water to total body water in the affected upper limb. The InBody S10 is a portable version of the Food and Drug Administration-approved multi-frequency bioelectrical impedance plethysmography body composition analyzer. It is highly reliable and reproducible for monitoring mild-to-moderate degree BCRL patients [17]. Patients were examined in the supine position, and eight electrodes were positioned (two for each foot and hand). Each evaluation was performed at the same time of the day.

A skinfold caliper (Cambridge Scientific Industries Inc., Cambridge, MD, USA) was used to measure the skin thickness (mm). The most fibrotic area in the forearm was identified through palpation and measured by skinfold caliper. The average of three measurements was used.

To check upper extremity function, patients completed the Quick Disabilities of the Arm, Shoulder, and Hand (QuickDASH) questionnaire. The QuickDASH is an 11-item questionnaire (e.g., open a tight or new jar, carry a shopping bag or briefcase, use a knife to cut food, etc.) that measures upper extremity-specific disability. Each item has five response options, and from the item scores, scaled scores are calculated, ranging from 0 (no disability) to 100 (most severe disability) [18].

### 2.4. Statistical Analysis

SPSS (version 22.0; IBM SPSS, Armonk, NY, USA) was used for the statistical analyses. The Mann–Whitney U test was used to compare baseline characteristics and test parameters test between the two groups. Statistical significance of differences between the pre-treatment, 3-week, and 3-month post treatments groups were determined by Wilcoxon signed-rank test. Statistical significance was defined as a *p*-value < 0.05.

## 3. Results

A total of 28 female patients with BCRL stage 2 lymphedema were enrolled and randomly assigned to the ESWT and control groups. None of the patients were lost to follow-up. All patients underwent radical mastectomy for breast cancer and received chemotherapy and/or radiation therapy. In the ESWT and control groups, the mean ages of patients were 57.51 ± 11.24 and 53.15 ± 8.59 years, and the mean lymphedema duration was 10.64 ± 5.33 and 14.5 ± 8.7 months, respectively (Table 1). There were no statistically significant differences between the two groups in terms of age, body mass index (BMI), lymphedema duration, types of chemotherapy and radiotherapy received, arm circumference, fluid volume, water ratio, QuickDASH score, and skin thickness (Table 1).

After 3 weeks of treatment, improvements were observed in both groups (Table 2). In the ESWT group, all the measured parameters showed statistically significant improvement at 3 weeks and 3 months post-treatment. However, only the circumference of the above elbow, elbow, below elbow, ratio of water content, and skin thickness showed statistically significant improvement in the control group measurements, 3 months post-treatment (Table 2).

Table 3 depicts the changes between the ESWT, and control groups measured at 3 weeks and at 3 months post-treatment. There were statistically significant differences (*p* < 0.001), in the circumference (cm) below the elbow, ratio of water content, and skin thickness (mm) between the ESWT group and the control group at both 3 weeks and 3 months.

The circumference below the elbow decreased from 25.57 ± 2.82 cm to 21.96 ± 2.67 cm in the ESWT group, and from 25.71 ± 3.30 cm to 25.45 ± 2.85 cm in the control group. The ratio of water content decreased from 0.39 ± 0.01 to 0.36 ± 0.01 in the ESWT group, and from 0.38 ± 0.01 to 0.37 ± 0.00 in the control group. The skin thickness decreased from 22.26 ± 5.43 mm to 18.39 ± 5.31 mm in the ESWT group, and from 23.58 ± 5.76 mm to 23.45 ± 5.74 mm in the control group (Table 2). No complications were observed in either of the groups during the study period.

## 4. Discussion

Lymphedema is caused by the accumulation of protein-rich interstitial fluid due to lymphatic dysfunction. Chronic lymphatic stasis also promotes infiltration of fibroblasts, adipocytes, keratinocytes, and neutrophils to the skin. This altered cellular and molecular environment in turn, promotes collagen deposition [19]. CDT is widely used as the primary treatment for lymphedema [20] and is regarded as the gold standard therapy. Szuba et al. reported that CDT was effective in reducing lymphedema volume by 45% [21]. The effectiveness of CDT has been proven at all stages, but t its effectiveness highly depends on the therapist’s skills and patient’s compliance [4,22,23]. In patients with advanced lymphedema with fibrotic change, the effectiveness of CDT is altered [19,24].

ESWT has been reported as an alternative treatment option for managing lymphedema. Previous studies indicate that ESWT promotes lymphangiogenesis and improves secondary lymphedema by activating vascular endothelial growth factor (VEGF) and fibroblasts [13,25]. In this study, it is considered that the improvement of lymphedema is attributed to lymphatic drainage as a result of lymphangiogenesis. Christ et al. reported that ESWT increases collagen synthesis and the number of elastic fibers and improves skin thickness and skin firmness [26]. Therefore, in this study, it is considered that the patients’ forearm fibrosis was improved by collagen synthesis and increase in the number of elastic fibers through ESWT. In a study by Bae et al., improvements in arm circumference, skin thickness, water volume, and pain score were reported when ESWT was administered to seven patients with stage 3 BCRL [14]. Similarly, in a study by Lee et al., edema and fibrosis improved without any significant side effects when ESWT was administered to patients with stage 2 BCRL with fibrosis [16]. Mehtap et al. also reported a decrease in the volume of lymphedema and an improvement in quality of life, when ESWT was applied to BCRL patients. They also noted that these effects persisted in the long term [27]. However, in that study, only volume reduction and improvement in the quality of life were observed, and detailed indicators, such as the differences in the circumference at various locations of the upper extremity and skin thickness, were not measured.

In our study, the below-arm circumference, ratio of water content, and skin thickness improved for up to 3 months when ESWT was administered in addition to CDT. An improvement in volume was observed in ESWT group. However, there was no statistically significant difference in the change of volume over time between the two groups, which is contrary to previously published RCTs and meta-analyses [15,16,28,29,30]. This lack of statistically significance difference may be attributed to the limited sample size of 28 patients and errors in measurement. Moreover, it is also possible that the combined improvements in below elbow circumference, skin thickness, and ratio of water content were not high enough to result in volume improvement of the entire arm. Several methods are employed for measuring the volume of lymphedema and monitoring the effectiveness of treatment. The most reliable and widely used method to measure lymphedema is volume measurement through immersion or volume measurement through circumferential measurement. Another method used to measure lymphedema volume is through bioelectrical impedance analysis measurement. However, lymphedema volume can also be measured through bioelectrical impedance analysis. One study reported that bioelectrical impedance analysis is an extremely sensitive and reliable technique for the early detection of lymphedema [31]. Other studies have used bioelectrical impedance analysis to diagnose lymphedema and reported that these results are accurate and enable therapeutic monitoring [32,33]. In this study, bioelectrical impedance analysis showed an improvement in lymphedema of the affected limb. The InBody S10 used for bioelectrical impedance analysis measurement in this study, is a multi-frequency bioelectrical impedance plethysmography body composition analyzer approved by the Food and Drug Administration. According to a study, which assessed the clinical feasibility of using InBody S10 in assessment and monitoring of BCRL, the ratio of water content (ratio of extracellular water to total body water) as measured by InBody S10, showed a positive correlation with circumference volume measurement [17]. In this study, bioelectrical impedance analysis showed a statistically significant improvement in ratio of water content compared to the control group, indicating an improvement in lymphedema. However, this improvement did not translate to an improvement in the quality of life. Moreover, as there is no established consensus on of how much difference between the values is clinically meaningful, this is considered a limiting point.

In ESWT, shock waves may either be focused or radial. In focused ESWT, shock waves deliver the maximum energy to the target area, whereas in radial ESWT, shock waves are pneumatically actuated to generate the maximum energy at the skin surface, which is then distributed radially to the tissues [34,35]. In this study, we utilized focused ESWT. The energy intensity in focused ESWT ranged from 0.001 to 0.5 mJ/mm^2^; a cut-off to 0.2 mJ/mm^2^ was used to classify the energy as being low or high [36]. Previous studies have reported that low-energy ESWT assists in tissue regeneration by increasing stem cell activity, promoting endothelial neovascularization, modulating inflammation, relieving pain and preventing soft tissue fibrosis [10,11,12]. Therefore, we utilized low-energy focused ESWT in this study. According to a systematic review and meta-analysis study published in 2021 [30], several RCTs were conducted by combining CDT and ESWT in BCRL. The study comprised a total of 8 studies, of which 4 used radial ESWT (rESWT) and 4 used focused ESWT (fESWT). All 4 studies which employed rESWT used energy intensity of 2 bar and frequency of 4 Hz. Among the studies that involved fESWT, two studies used intensity range of 0.040–0.069 mJ/mm^2^, one study used 0.056–0.068 mJ/mm^2^, and another study used 0.1 mJ/mm^2^. Additionally, two studies used frequency of 4 Hz, and one study used frequency of 5 Hz. Another study did not indicate frequency. The ESWT duration varied from 3 to 8 weeks, and the total ESWT sessions varied from 6 to 16 sessions [15,16,27,28,29,37,38,39]. In this study, fESWT was used, the energy intensity was 0.056–0.068 mJ/mm^2^, the frequency was 4 Hz, and 9 sessions were conducted 3 times a week, for a total of 3 weeks.

This study had several limitations. First, not all patients were identified as having lymphedema by lymphoscintigraphy. The classification of lymphedema stages based on the International Society of Lymphology Executive Committee (ISL) consensus document used in this study can be subjective. Second, using the QuickDASH score, improvement in lymphedema did not translate to an improvement in a patient’s quality of life. In addition to the QuickDASH score, other indicators should be utilized to evaluate the quality of life. Third, the sample size was relatively small; therefore, studies with a larger number of patients are needed. Fourth, this study measured the outcomes after 3 months to represent a long-term effect. Considering the possibility of follow-up loss of patients and individual differences in the long-term maintenance phase, a follow-up period of 3 months was selected. Fifth, factors such as dexterity and occupation that can affect the response to treatment or stage could not be checked in all patients is another limitation of this study. However, as described above, lymphedema is a chronically progressive disease; therefore, a long-term study is needed to determine whether the effects of ESWT last longer than 3 months. In addition, further studies are needed to determine the type, intensity, frequency, and total sessions of ESWT.

Although several studies have reported the short-term effects of ESWT in patients with BCRL, long-term effects of ESWT remains understudied. This study, therefore, provides meaningful insights that have been reported only in a few previous studies.

## 5. Conclusions

ESWT improved lymphedema in stage 2 BCRL patients, evidenced by a decrease in upper extremity circumference, ratio of water content, and skin thickness. Additionally, these effects were confirmed to persist for at least 3 months post-treatment. Along with CDT, ESWT may be an additional treatment modality for patients with lymphedema.

## Figures and Tables

**Table 1:** Baseline characteristics of subjects.

	ESWT Group (n = 14)	Control (n = 14)	*p*-Value
Age (yr)	57.51 ± 11.24	53.15 ± 8.59	0.59
Female	14	14	-
BMI (kg/m^2^)	24.87 ± 2.98	26.4 ± 5.18	0.51
Lymphedema duration (mo)	10.64 ± 5.33	14.5 ± 8.7	0.246
Received chemotherapy	13	14	0.769
Received radiotherapy	11	13	0.769
Circumference (cm)			
Above elbow	28.14 ± 3.86	28.4 ± 3.73	0.91
Elbow	25.89 ± 2.48	26.11 ± 2.97	0.91
Below elbow	25.57 ± 2.82	25.71 ± 3.30	0.95
Wrist	16.6 ± 1.28	16.3 ± 1.67	0.57
Metacarpophalangeal joints	17.8 ± 1.42	17.9 ± 1.23	0.95
Volume (mL)	877.86 ± 131.98	890 ± 154.07	0.91
Ratio of water content	0.39 ± 0.01	0.38 ± 0.01	0.09
QuickDASH score	4.29 ± 6.82	3.76 ± 6.35	0.95
Skin thickness (mm)	22.26 ± 5.43	23.58 ± 5.76	0.73

ESWT, extracorporeal shock wave therapy; BMI, body mass index; QuickDASH, Quick Disabilities of the Arm, Shoulder, and Hand. Values are mean ± standard deviation.

**Table 2:** Comparison between pre-treatment and post-treatment measurements in both groups.

		ESWT Group				Control Group		
	3 Weeks	*p*-Value	3 Months	*p*-Value	3 Weeks	*p*-Value	3 Months	*p*-Value
Circumference (cm)								
Above elbow	27.6 ± 3.48	0.027 *	27.3 ± 3.61	0.001 *	28.4 ± 3.47	0.185	28.1 ± 3.61	0.008 *
Elbow	25.5 ± 2.3	0.015 *	25.4 ± 2.48	0.013 *	25.9 ± 2.58	0.125	25.7 ± 2.57	0.034 *
Below elbow	23.6 ± 2.59	0.001 *	21.96 ± 2.67	0.001 *	25.8 ± 2.66	0.669	25.5 ± 2.85	0.02 *
Wrist	16 ± 1.3	0.005 *	16.2 ± 1.27	0.018 *	16 ± 1.4	0.458	15.9 ± 1.41	0.063
Metacarpophalangeal joints	17.4 ± 1.5	0.034 *	17.3 ± 1.46	0.022 *	17.6 ± 1.65	0.252	17.5 ± 1.39	0.124
Volume (mL)	845.71 ± 124.39	0.006 *	832.14 ± 122.17	0.001 *	875 ± 136.82	0.454	869.64 ± 125.27	0.563
Ratio of water content	0.37 ± 0.00	0.001 *	0.36 ± 0.01	0.001 *	0.38 ± 0.00	0.067	0.37 ± 0.00	0.009 *
QuickDASH score	3.37 ± 5.34	0.028 *	3.03 ± 4.77	0.028 *	3.6 ± 5.8	0.485	3.54 ± 5.79	0.225
Skin thickness (mm)	19.22 ± 5.22	0.001 *	18.39 ± 5.31	0.001 *	23.53 ± 5.83	0.608	23.45 ± 5.74	0.045 *

ESWT, extracorporeal shock wave therapy; QuickDASH, Quick Disabilities of the Arm, Shoulder, and Hand. Values are mean ± standard deviation. * *p* < 0.05 by Wilcoxon signed-rank test.

**Table 3:** Comparison of changes between two groups at 3 weeks and 3 months post-treatment.

	3 Weeks			3 Months		
	ESWT	Control	*p*-Value	ESWT	Control	*p*-Value
ΔCircumference (cm)						
ΔAbove elbow	0.5 ± 0.85	0.21 ± 0.80	0.804	0.81 ± 0.86	0.51 ± 0.53	0.454
ΔElbow	0.36 ± 0.41	0.25 ± 0.64	0.635	0.49 ± 0.56	0.44 ± 0.64	0.982
ΔBelow elbow	1.93 ± 0.43	0.11 ± 0.81	<0.001 *	3.61 ± 0.63	0.26 ± 0.73	<0.001 *
ΔWrist	0.39 ± 0.35	0.14 ± 0.86	0.058	0.39 ± 0.50	0.36 ± 0.72	0.454
ΔMetacarpophalangeal joints	0.43 ± 0.65	0.29 ± 0.78	0.874	0.48 ± 0.68	0.32 ± 0.46	0.635
ΔVolume (mL)	32.14 ± 53.23	15 ± 76.74	0.178	45.71 ± 61.86	20.36 ± 90.27	0.137
ΔRatio of water content	0.016 ± 0.006	0.002 ± 0.004	<0.001 *	0.03 ± 0.009	0.003 ± 0.003	<0.001 *
ΔQuickDASH score	0.91 ± 1.83	0.16 ± 0.66	0.114	1.26 ± 2.52	0.23 ± 0.64	0.352
ΔSkin thickness (mm) *	3.04 ± 0.5	0.05 ± 0.41	<0.001 *	3.87 ± 0.54	0.13 ± 0.77	<0.001 *

ESWT, extracorporeal shock wave therapy; QuickDASH, Quick Disabilities of the Arm, Shoulder, and Hand. Values are mean ± standard deviation. * *p* < 0.05 by Mann–Whitney U test.

## Data Availability

Not applicable.
